# 770. Impact of COVID-19 Pandemic on Central Line-associated Bloodstream Infections in Metropolitan Detroit

**DOI:** 10.1093/ofid/ofab466.967

**Published:** 2021-12-04

**Authors:** Geehan Suleyman, Nicholas sturla, Smitha Gudipati, Indira Brar, Ramesh Mayur

**Affiliations:** 1 Henry Ford Hospital, Detroit, Michigan; 2 Henry Ford Health System, Detroit, Michigan

## Abstract

**Background:**

Recent publications suggest that central line-associated bloodstream infection (CLABSI) rates have increased in US hospitals during the COVID-19 pandemic. The objective of this study was to evaluate the impact of COVID-19 pandemic on CLABSIs.

**Methods:**

This was a retrospective cross-sectional study comparing CLABSI rate per 1,000 central line (CL) days, blood culture (BC) utilization rate per 1,000 CL days, CL utilization rate per 1,000 patient days, Standardized Infection Ratio (SIR) and Standardized Utilization Ratio (SUR) in the pre-COVID-19 period from January 1, 2019 to December 31, 2019 to the COVID-19 period from April 1, 2020 to March 31, 2021 at an 877-bed tertiary care hospital in Detroit, Michigan. CLABSI, and BC and CL utilization rate were extracted from the electronic medical record (Epic™ Bugsy). SIR and SUR data were extracted from National Healthcare Safety Network (NHSN).

**Results:**

The average CLABSI rate per 1,000 CL days increased 24% from 1.66 to 2.06. Twenty percent of patients were hospitalized for COVID-19. The BC utilization rate per 1,000 CL days decreased from 0.43 to 0.32 with a 26% reduction. However, CL utilization increased by 28% from 0.25 to 0.32 (Figure 1). However, CLABSIs due to common commensals decreased from 13.8% to 10.9%. The SIR increased significantly from 1.055 to 1.795 (P-value 0.008), resulting in a 70% increase. The overall SUR also increased from 0.900 to 0.988 (P-value < 0.001). Figure 2 is a control chart of the CLABSI rate from July 2019 to April 2021.

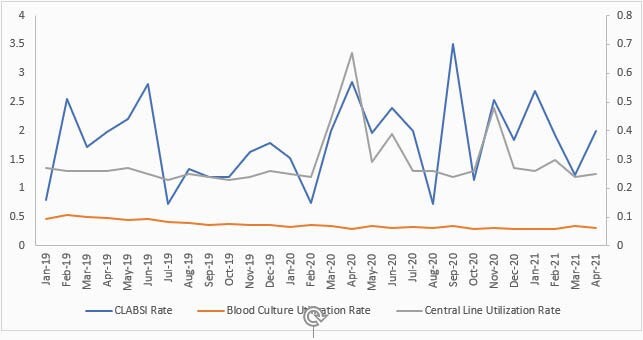

Figure 1. CLABSI, blood culture utilization and central line utilization rates pre-and during COVID-19 pandemic

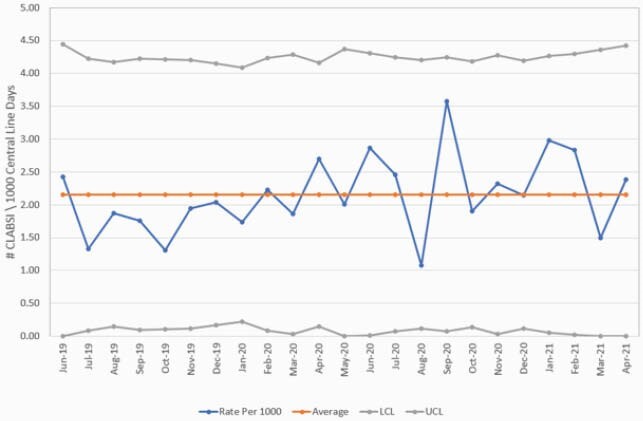

Figure 2. CLABSI control chart pre-and during COVID-19 pandemic

**Conclusion:**

During the COVID-19 pandemic, there was a significant increase in CL utilization, CLABSI rate, SIR and SUR likely due to higher acuity in COVID-19 patients despite a decrease in BC orders.

**Disclosures:**

**All Authors**: No reported disclosures

